# Syllabus of the Treatment of Fractures of the Cranium

**Published:** 1884-11

**Authors:** 


### SYLLABUS OF THE TREATMENT OF FRACTURES OF THE CRANIUM.

#### SIMPLE FISSURED FRACTURES.

1. No evident depression, no brain symptoms. No operation.
2. No evident depression, with brain symptoms. Incise scalp and trephine.
3. With evident depression, no brain symptoms. Incise scalp and possibly trephine.
4. With evident depression, with brain symptoms. Incise scalp and trephine.

#### SIMPLE COMMINUTED FRACTURES.

5. No evident depression, no brain symptoms. Incise scalp and probably trephine.
6. No evident depression, with brain symptoms. Incise scalp and trephine.
7. With evident depression, no brain symptoms. Incise scalp and trephine.
8. With evident depression, with brain symptoms. Incise scalp and trephine.

#### COMPOUND FISSURED FRACTURES.

9. No evident depression, no brain symptoms. No operation, but treat wound.
10. No evident depression, with brain symptoms. Trephine.
11. With evident depression, no brain symptoms. Possibly trephine.
12. With evident depression, with brain symptoms. Trephine.

COMPOUND COMMINUTED FRACTURES.

13. No evident depression, no brain symptoms. Probably trephine.

14. No evident depression, with brain symptoms. Trephine.

15. With evident depression, no brain symptoms. Trephine.

16. With evident depression, with brain symptoms. Trephine.

PUNCTURED AND GUNSHOT FRACTURES.

17. In all cases and under all circumstances, trephine.

In classes 3 and 11, I should be inclined to trephine if the depression was marked, or the fissures sufficiently multiple to approach the character of a comminuted fracture.

In classes 5 and 13, I should trephine, unless the comminution was found to be inconsiderable.

The operation, when decided upon, should be performed at once, or certainly not delayed more than a few hours.

All cases, whether trephined or not, should be treated as cases of incipient inflammation of the brain.

---

SIR JOSEPH LISTER writes to the *British Med. Journal* that one drachm by weight of a solution of one part of corrosive sublimate in one and a half parts of glycerine contains two-fifths its weight, or twenty-four grains of the sublimate. This, multiplied by 1,000 (the proportion of water required), gives 24,000 grains, which is very nearly three pints. It is, however, much more convenient to use fluid measure than weight, and a fluid drachm of the glycerine solution referred to requires four pints of water to produce the 1 to 1,000 solution.

---

A PRIVATE letter from Prof. W. H. Heath, written from London, states that he expects to sail for home Nov. 4th, with health so much improved that he will resume his duties in the Hospital Marine Service and in the Niagara Medical College. This information will gratify the numerous friends of our esteemed colleague, both here and elsewhere.

